# *In-vivo *imaging of retinal nerve fiber layer vasculature: imaging - histology comparison

**DOI:** 10.1186/1471-2415-9-9

**Published:** 2009-08-23

**Authors:** Drew Scoles, Daniel C Gray, Jennifer J Hunter, Robert Wolfe, Bernard P Gee, Ying Geng, Benjamin D Masella, Richard T Libby, Stephen Russell, David R Williams, William H Merigan

**Affiliations:** 1 University of Rochester Eye Institute, University of Rochester, Rochester, NY, USA; 2 Center for Visual Science, Institute of Optics, University of Rochester, Rochester, NY, USA; 3 Department of Ophthalmology and Visual Sciences, University of Iowa, Iowa City, IA, USA

## Abstract

**Background:**

Although it has been suggested that alterations of nerve fiber layer vasculature may be involved in the etiology of eye diseases, including glaucoma, it has not been possible to examine this vasculature *in-vivo*. This report describes a novel imaging method, fluorescence adaptive optics (FAO) scanning laser ophthalmoscopy (SLO), that makes possible for the first time *in-vivo *imaging of this vasculature in the living macaque, comparing *in-vivo *and *ex-vivo *imaging of this vascular bed.

**Methods:**

We injected sodium fluorescein intravenously in two macaque monkeys while imaging the retina with an FAO-SLO. An argon laser provided the 488 nm excitation source for fluorescence imaging. Reflectance images, obtained simultaneously with near infrared light, permitted precise surface registration of individual frames of the fluorescence imaging. *In-vivo *imaging was then compared to *ex-vivo *confocal microscopy of the same tissue.

**Results:**

Superficial focus (innermost retina) at all depths within the NFL revealed a vasculature with extremely long capillaries, thin walls, little variation in caliber and parallel-linked structure oriented parallel to the NFL axons, typical of the radial peripapillary capillaries (RPCs). However, at a deeper focus beneath the NFL, (toward outer retina) the polygonal pattern typical of the ganglion cell layer (inner) and outer retinal vasculature was seen. These distinguishing patterns were also seen on histological examination of the same retinas. Furthermore, the thickness of the RPC beds and the caliber of individual RPCs determined by imaging closely matched that measured in histological sections.

**Conclusion:**

This robust method demonstrates *in-vivo*, high-resolution, confocal imaging of the vasculature through the full thickness of the NFL in the living macaque, in precise agreement with histology. FAO provides a new tool to examine possible primary or secondary role of the nerve fiber layer vasculature in retinal vascular disorders and other eye diseases, such as glaucoma.

## Background

Although subjective threshold visual field testing and stereoscopic biomicroscopy of the disc and disc photos provide powerful clinical assessments of eye disorders that affect retinal ganglion cells, quantitative, objective measurement of NFL thickness has added an additional diagnostic indicator of such disorders [1,2] and is routinely used in monitoring response to therapy. The mechanism by which NFL thinning develops, however, is poorly understood. Epidemiologic, clinical and laboratory evidence supports the contention that nerve fiber layer damage results from vascular compromise or dysregulation, probably within the lamina cribrosa [3], focussing attention on the highly specialized vasculature that supplies the nerve fiber layer [4,5], known as the radial peripapillary capillaries (RPCs) [6]. Prior reports have suggested that these capillaries might be involved in the etiology of glaucoma [6], although others have pointed out that it would be difficult to establish cause and effect between nerve fiber layer thinning and disappearance of the NFL vasculature [7]. Although the RPCs are unlikely to represent a primary site of disease vulnerability, they may reflect NFL or contiguous disc capillary network compromise. A better understanding of these factors will be important in understanding NFL clinical and optical coherence tomographic (OCT) data, and may provide a supplementary indicator of ganglion cell loss [8].

Evaluation of RPCs in glaucoma and other eye diseases has been limited because of the difficulties of imaging these vessels using conventional fluorescein or indocyanine green angiography. However, as reported here, we can robustly image these vessels and determine their depth with high precision in macaque monkeys using *in vivo *adaptive optics fluorescent imaging. RPC vessels are thin and form arrays that are oriented parallel to the course of nerve fiber bundles [4,6,9]. As the axial focus of the imaging shifts toward greater retinal depth, the appearance of the vessels changes from parallel orientation to the polygonal and more random pattern that is typical of retinal vasculature [9]. Since the thickness of tissue supplied by RPCs is of great interest for evaluating the effect and potential participation in glaucomatous NFL changes, we compared *in vivo *to *ex vivo *thickness of the RPCs in the same eye and found strong correlation of RPC pattern within the NFL.

## Methods

### Subjects

Two juvenile macaque monkeys (Macaca mulatta) 6.3 and 5.5 kg, served as subjects. All experimental protocols were approved by the University Committee of Animal Resources at the University of Rochester Medical Center, complied with the Public Health Service policy on Humane Care and Use of Laboratory Animals, and adhered to the ARVO Statement for the Use of Animals in Ophthalmic and Vision Research. Axial lengths of the imaged eyes were measured with an IOLMaster (Carl Zeiss Meditec, Jena, Germany) (5 determinations for each eye, standard deviation approximately 3% of mean) in the anesthetized monkey, and used to calculate transverse and axial retinal distances. Each monkey was fitted with a rigid, gas permeable contact lens to preserve the corneal epithelium and reduce astigmatism and spherical refractive error. Standard ophthalmic trial lenses were used to reduce residual astigmatism and spherical error to values that could be corrected with the AO deformable mirror.

### In-vivo imaging

**Figure 1:**
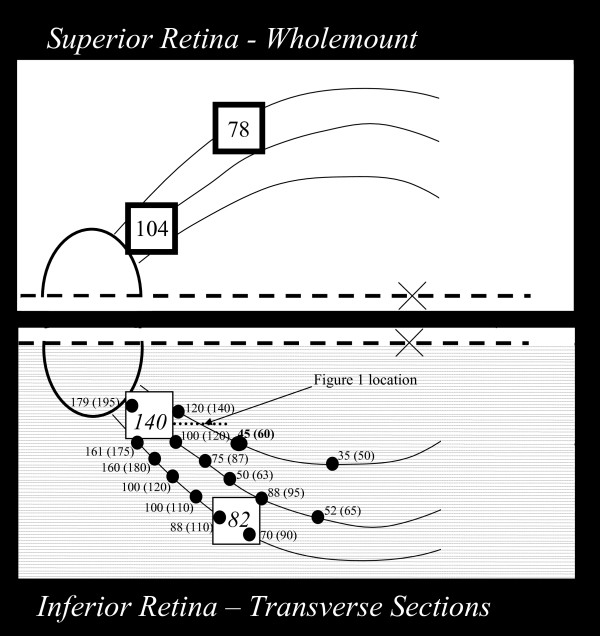
**Map of the location in the retina of monkey 1 where *in-vivo *images (heavy border squares) and RPC network thickness measures (dots) were made**. The superior retina was prepared as a wholemount, but because this preparation compressed the tissue no measurements of radial peripapillary bed (RPC) or nerve fiber layer (NFL) thickness were made in this location. The lower retina was sectioned (fine dotted lines) parallel to the horizontal raphe (heavy dashed line). The ellipse shows the location of the optic disc and "x" represents the foveal location. Curving lines show the course of nerve fibers identified from fundus images. Numerical values for RPC network thickness and NFL thickness (in parentheses) were measured with *ex-vivo *confocal microscopy. *In-vivo *FAOSLO measures of thicknesses of the RPC network are shown in larger font numbers. RPC thickness in corresponding locations (thin border squares) in the lower retina are shown in italics. The heavy dotted line shows the location of the sections illustrated in Figure 2.

The fluorescence adaptive optics scanning laser ophthalmoscope (FAO-SLO) instrument, monkey imaging methods, and the post-processing methods have been previously described [10]. For *in-vivo *imaging, the monkeys were anesthetized with isoflurane at a dosage (typically 2%) sufficient to minimize large ocular movements and eliminate microsaccades. Vital signs and body temperature were continuously monitored. The monkey retinas were imaged *in-vivo *with the AO system using reflected light at 794 nm ± 17 nm to generate a high-resolution fundus photograph of the peripapillary region. Two regions of interest were selected (along the temporal arcuate NFL, see Figure 1), and 1000 frames were captured at each focal plane in a series of focus depths. We used a deformable mirror to focus through the retina at 0.1 D steps from the inner retinal surface, above the nerve fiber layer, to the pigmented epithelium. The Elmsley model eye was used to convert diopters of focus (F) to axial distance using the following formula: microns of focus = 4/3 [1/[power(D) +focus(D)]-1/power(D)]×10^6^, where P is the power of the eye in diopters, calculated by linearly scaling eye size by the measured axial length assuming an emmetropic eye. For the monkeys used in this study, 0.1D corresponded to approximately 26 μm.

#### Fluorescein Injections

In each imaging session we used two 0.5 mL intravenous injections of sodium fluorescein (scaled to be equivalent to the standard human dose in ml/body weight) to enhance the contrast of the vasculature at the locations of interest. We obtained a reflectance video with 794 nm light and simultaneously used 488 nm laser excitation and recorded the emitted fluorescein fluorescence at 520 nm ± 35 nm. Each region of interest (ROI) was imaged at depths ranging from the NFL to the RPE in 0.1D steps. The detector gain was continually optimized to minimize the number of saturated pixels in the image. Because of the weak fluorescence signal from the retina, 1000 raw video frames were dual-registered and averaged using methods described previously [10]. (Although high signal/noise was present in single frames obtained in the first few seconds after fluorescein injection, registration and averaging allowed us to obtain high quality images up to 30 minutes after injections, despite the faint fluorescence present at this time). Single frame and registered/averaged frame results are similar in human subjects.

### Ex-vivo preparation

The monkeys were euthanized for histological analysis. Under deep anesthesia, both monkeys were perfused, initially with 2 liters of saline to flush blood from the vascular system and then with two liters of 4% paraformaldehyde, infused over a 1 hour period. The eyes were fixed further in the eyecup for an additional 40 minutes. The retinas were cut in half along the horizontal raphe, and relief cuts were made in each section. The two *in-vivo *imaging areas, located in the superior retina were preserved in one tissue block. The superior retinal ROI was simultaneously incubated with three primary antibodies, one which binds to neurofilaments (rabbit polyclonal neurofilament 200, Chemicon, Temecula, CA) and the other to blood vessel endothelium (mouse monoclonal CD31, and mouse monoclonal von Willebrandts factor, Lab Vision, Fremont, CA). Dye-coupled secondary antibodies were applied to label the neurofilaments with Alexa 555 (goat anti-rabbit (H&L)) and the vessels with Alexa 488 (goat anti-mouse (H&L)) (Invitrogen, Carlsbad, CA). Then the superior retinal tissue was removed from solution, placed as a flat mount on a slide, covered in Vectashield (Vector, Burlingame, CA) to minimize fading of fluorescence, and cover slipped. The inferior retinal tissue was embedded in agar and sectioned (transverse sections parallel to the horizontal raphe) at 60 μm thickness on a vibratome (Microm, Walldorf, Germany). Every third section was reacted with the same antibodies as above, followed by dye-coupled secondary antibodies to label the neurofilaments with Alexa 543 and the vessels with Alexa 488, as well as 4', 6-diamino-2-phenylindole, dihydrochloride (DAPI) (Invitrogen, Carlsbad, CA) to identify nuclei of retinal neurons. The sections were covered in Vectashield and coverslipped.

### *Ex-vivo *imaging

*Ex-vivo *wholemount and transverse sections were imaged on a Zeiss 510 Meta confocal microscope using a 10× air objective with an NA of 0.3, an NA similar to that used to perform *in-vivo *FAO SLO. Six to ten 10× imaging fields were needed to visualize all areas of each section. The images were digitally combined to create one image per tissue section. The retinal location of the digitally assembled sections were registered against a color fundus photograph using vascular landmarks.

### *In-vivo *and *ex-vivo *measurements

**Figure 2:**
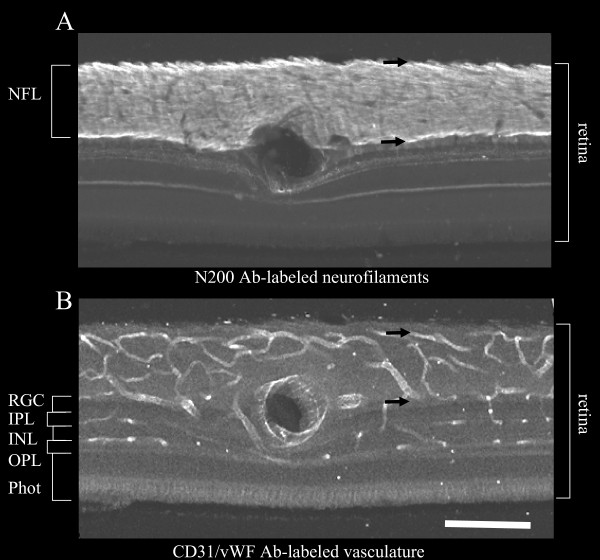
**Epi-fluorescence micrographs of adjacent retinal sections from the inferior retina of monkey 1 (see Figure 1 for location) labeled for (A) axons-neurofilament - N 200 and (B) vasculature -CD31/vWF**. The RPC and nerve fiber layer thickness shown in the lower half of Figure 1 were measured in such sections as illustrated in Figure 2 by the distance between the black arrows. Scalebar indicates 150 μm.

Figure 2 illustrates the method that was used to measure the thickness of the nerve fiber and vascular layers in the retina at the locations indicated by large dots in Figure 1. Vessel caliber was also measured on these sections. Pairs of sections were dual-labeled for NF 200 and CD-31/Von Willenbrandt's factor. Because the fluorescence tags of the secondary antibodies (Alexa 543 and Alexa 488, respectively) differ in emission peak, we simultaneously recorded total fluorescence on a confocal microscope and separated fluorescence channels in post processing with ImageJ. Thus the images remain in registration throughout the analysis. Section A (Figure 2) portrays the neurofilament label, the intensely labeled NFL and other retinal layers with light labelling. Section B (Figure 2) portrays vessel labelling, showing the luminal border of retinal vascular endothelial cells.

a. RPC thickness-

**Figure 3:**
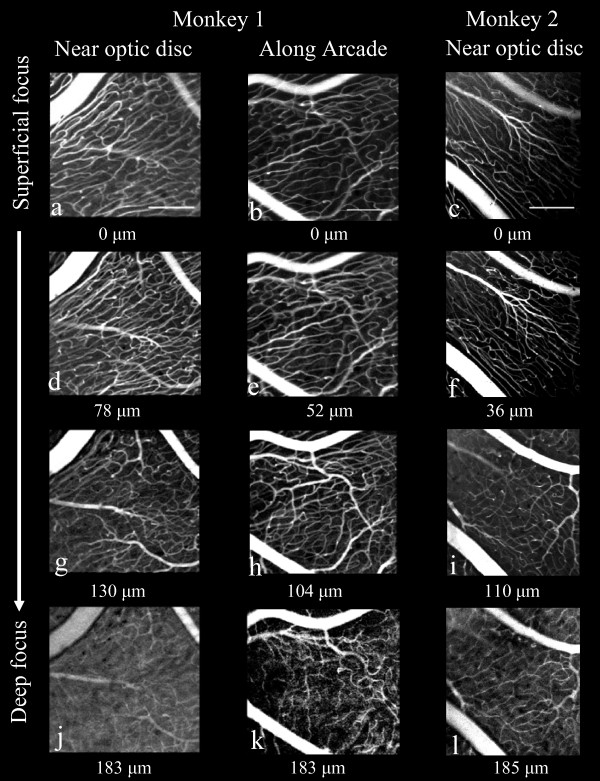
**Through-focus series of *in-vivo *adaptive optics images of RPCs from two retinal locations in monkey 1 and one retinal location in monkey 2**. One location in both monkeys is near the optic disc; monkey 1 (a, d, g, j) and monkey 2 (c, f, i, l) and the second is 5 mm temporal to the optic disc of monkey 1(b, e, h, k) (see Figure 1 for locations in monkey 1). At a focal plane deep within the NFL, a transition was found from RPC to to the typical, polygonal ganglion cell layer/retinal circulation (j, k, l). The focus depth set by the deformable mirror relative to the most superficial vasculature is displayed under each image. Scalebars indicate 200 μm.

*in-vivo *- Thickness of the RPC bed was measured fromadaptive optics images, some of which are illustrated in Figure 3. The inner border of the RPC bed was taken as the first image which showed RPCs clearly and the outer border as the first image which showed the vascular pattern typical of the ganglion cell layer. Uncertainty in this measurement reflects the depth sampling interval (approximately 26 μm) as well as the estimate of which vessels were near the center of focus. We also measured maximal RPC thickness in four quandrants of each image and averaged the four measures.

*ex-vivo *- RPC thickness was determined by measurements on transverse sections, as illustrated in Figure 2B, of the distance from the most superficial (vitread) NFL vessels to the outermost RPC boundary, which is determined as the inflection in pattern between the extremely long capillaries with thin walls, little variation in caliber and parallel-linked structure oriented parallel to the NFL axons, and the polygonal pattern typical of the ganglion layer and retinal vasculature. The outer (sclerad) border of the RPC bed was also correlated with the location of the most superficial ganglion cell dendrites, which were highly visible due to neurofilament labelleing. These patterns were easily distinguishable within *in-vivo *images as well as histologic transverse sections. This measurement is illustrated in Figure 2B as the distance between the two black arrows on a section close to the optic nerve head whose location is shown in Figure 1. Each *ex-vivo *result represents the average of five measurements over a 50 micron region centered at each data point.

b. NFL thickness - NFL thickness was determined by measuring neurofilament label in transverse sections as illustrated in Figure 2A. Each result represents the average of five measurements over a 50 micron region centered at each data point. NFL thickness could not be measured from *in-vivo *imaging data.

c. vessel pattern - the vessel pattern within versus beneath the RPC bed was determined by appearance (RPCs, figure 3a, b, c, d,, and 3f) and (ganglion cell vasculature, figure 3g, h. and 3i).

d. vessel diameter - was measured at 20 randomly chosen locations on each of the 6 *in-vivo *AOSLO RPC images illustrated in Figure 3(a, b, c, d, e), and 3f using the open-source NIH software package, ImageJ. Twenty (20) random x, y locations were generated on each image and a measurement was taken of the caliber of the nearest RPC vessel by drawing a line segment across the vessel, whose length was registered by the software. Similar measures were made at 20 randomly chosen locations on each digital image from 6 sections from the lower retina of monkey 1, 3 sections at each of the two locations that correspond to the *in-vivo *imaging of the upper retina.

## Results

Figure 1 diagrammatically represents the relative locations in the retina of monkey 1 from which *in-vivo *FAO-SLO and *ex-vivo *confocal microscopy images of RPCs were taken, as well as the corresponding thickness measurements. The two heavy border squares in the superior retinal block represent the locations of *in-vivo *imaging in this monkey, and the respective measured average RPC thickness calculated from the *in-vivo *images is indicated within each box.

### RPC thickness

*In-vivo *- From *in-vivo *FAO-SLO fluorescein angiography, the thickness of the superficial parallel-linked layer of RPC vasculature was measured as 104 μm near the optic disc and 78 μm at the location 5 mm along the arcade (heavy squares in superior retina) as shown in Figure 1.

*Ex-vivo *- Thickness measurements of RPC bed were also made from transverse sections in the inferior retinal block, diagrammed in Figure 1, and are shown as the first value (before parentheses) adjacent to the marked location at which they were made (heavy dots). For comparison with the thickness measures made from *in-vivo *images, RPC network thickness measurements were also made (average of four points within each square) at the locations in inferior retina (light squares) that correspond in eccentricity to the *in-vivo *imaged locations. Thickness measurements are not reported for the imaged locations in the whole-mounted tissue because of tissue compression presumably caused by pressure of the coverslip.

Comparison of *in-vivo *to *ex-vivo *measures - RPC thickness measured *in-vivo *was 104 and 78 μm at the locations shown in Figure 1 closer to and more distant from the optic nerve. Comparison measures from comparable locations in inferior retina were 140 and 82 μm, indicating comparable values for the two methods on the same retinas.

### NFL thickness

NFL thickness measures are shown in parentheses in Figure 1 at each location indicated by dots at which RPC bed thickness was measured. In all cases, the NFL thickness was 10 to 20 μm thicker than the RPC bed, reflecting the absence of RPCs in the most superficial portion of the NFL.

### Vessel pattern

**Figure 4:**
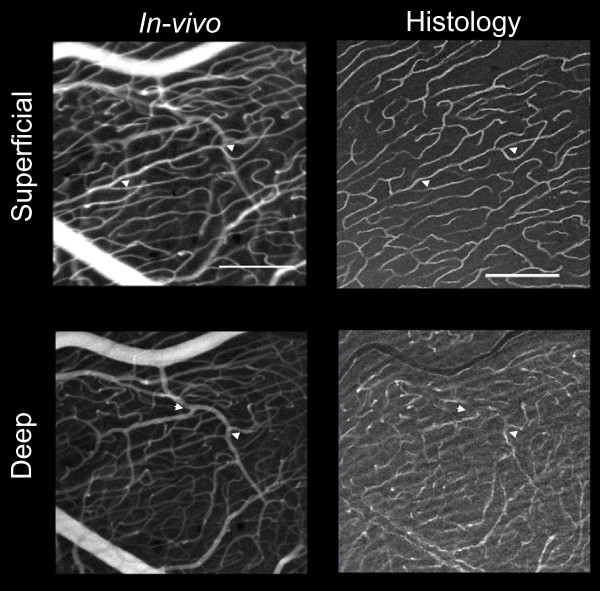
**Comparison of *in-vivo *imaging and *ex-vivo *immunohistochemistry at two depths for images taken 5 mm temporal to the optic disc in monkey 1**. Vessel branch points that can be seen in both the AO image and microscopy are indicated by arrowheads. Scalebars indicate 200 μm.

Figure 3 shows examples from a through-focus series of *in-vivo *adaptive optics images of radial peripapillary capillaries at two locations in monkey 1, near the maximum of nerve fiber layer thickness near the optic disc (A), and at a location about 5 mm from the optic disc (B), where the nerve fiber layer thickness is much narrower. The locations of these two imaged regions are shown to scale on the map of the retina of monkey 1 in Figure 1 (heavy squares). An additional location near the optic disc is shown in Figure 3 for the second monkey. These images illustrate the two important patterns of vessels identified by *in-vivo *imaging. First, there is an abrupt transition from a superficial parallel-linked architecture of extraordinarily long, uniformly narrow vessels (capillaries) oriented parallel to nerve fiber bundles, to a deeper polygonal architecture of more varied diameter vessels typical of the ganglion cell layer/retinal vasculature. The parallel-linked, long capillaries were found throughout the full thickness of the NFL (Figure 1, D, E, F) with the exception of the most superficial 10 - 20 μm. Second, in both the FAO-SLO images and in the microscopy superficial (inner) vessels show the highest contrast, while those deep (outer) in the retina show reduced contrast. The transverse separation of RPC vessels in *ex-vivo *measures was approximately 20 μm. The distinction between RPC and RGC vasculature can also be seen in Figure 4, which compares *in-vivo *FAO-SLO fluorescein-imaged vessels and *ex-vivo *antibody-imaged confocal microscopy of the retinal wholemounted tissue. There was precise correspondence of vessel pattern in the comparative images. The *in-vivo *imaging involves a larger depth of focus, explaining the simultaneous visibility of several vascular layers. We found a consistent gap between RPC vessels and large retinal vessels that was approximately 50 μm. (see figure 4 lower right).

### Vessel diameter

*In-vivo *and *ex-vivo *vessel diameter measurements are shown in Table 1. Mean *in-vivo *diameter was 5.12 and mean *ex-vivo *diameter was 5.03, not statistically different on an unpaired t test (P = 0.78).

**Table 1:** RPC vessel diameter

	**in-vivo**		**ex-vivo**	
Figure	Mean	SD	Mean	SD

3a	5.9	1.0	5.9	1.0
3b	5.0	1.4	5.2	0.9
3c	5.0	1.0	5.0	1.4
3d	5.2	0.9	4.5	0.82
3e	4.5	0.82	5.1	1.0
3f	5.3	1.2	4.5	0.7

## Discussion

This study demonstrates precise *in-vivo *quantification of the circulations of the NFL and retina, including features of the RPC vascular bed, which match *ex-vivo *images of the same tissue made by confocal microscopy. The inner or superficial RPCs have a distinctive and easily recognizable structure of parallel-linked, long uniform-diameter vessels that are oriented parallel to the nerve fiber bundles. The transition from RPCs to typical retinal/ganglion cell layer vasculature is abrupt and roughly corresponds to the bottom of the NFL (outer margin). The entire RPC network remains within the NFL, but its network thickness is consistently 10 to 20 μm thinner than the NFL. These results underscore the coupling of NFL and the RPCs. This relationship can now be imaged in human glaucoma patients to determine how RPCs and their networks may change with the development of glaucomatous NFL hemorrhages and thinning [e.g. [11]].

These findings also support the notion of a linked spatial and functional relationship between the NFL and the RPCs. Evaluation of disturbances of NFL/RPC density or distribution *in-vivo *are now possible for normal and diseased eyes. One clinically important, but poorly understood, phenomenon which could be functionally evaluated with this method is the development of disc hemorrhages in glaucoma. The superficial distribution, recurrent nature, and distance between hemorrhage and optic disc, suggest that RPCs are the source of the blood. However, without longitudinal (i.e. in vivo) evaluation of the association of disc hemorrhage with glaucoma progression will not be determined.

### Fidelity with which *in vivo *imaging captured the three dimensional structure of RPCs and their network

We have previously shown that the transverse resolution of reflectance AO is approximately 1.6 μm [12], thus it captures even subtle features of RPC vessels, which are approximately 5 μm in diameter. In comparing *in-vivo *FAO-SLO fluorescein angiography images to 10, 20 and 40× *ex-vivo *confocal images of wholemounts of the same regions we found that the *in-vivo *images could identify every feature and dimension of every vessel in the *ex-vivo *images. This was particularly useful when identifying portions of vessels that extended beneath other vessels. RPC bed thickness measured in-vivo and ex-vivo could be compared at the locations close to the optic disc (140 μm ex-vivo versus 104 μm in-vivo) and farther from the optic disc (82 μm ex-vivo versus 78 μm in-vivo) This fidelity reflected the confocal design of the AO system, whose resolution permits the determination of axial position with an accuracy of approximately 6.5 μm [12]. We found that RPC vessels were axially spaced on average approximately 20 μm apart, with no layering evident, and that each vessel was visible in the through-focus series. The thickness of the RPC layer could not be directly compared between *in-vivo *imaging and microscopy, because the wholemount preparation slightly decreased the thickness of RPC and NFL. However, comparison to the RPC network thicknesses measured from sections of the inferior retina at equal eccentricities showed close correspondence to *in-vivo *measurements. The major difference between the RPC networks at the two retinal regions of interest was network thickness. There was no apparent difference in the diameter or distance between RPCs. It is worth noting that the thinnest NFL was measured along the horizontal raphe. Even at this location, where NFL might be expected to be thin, the NFL exceeded 20 μm, consistent with the observation that upper and lower hemisphere fibers mix across the horizontal raphe [13].

### Comparison to previous histological measures of RPC and NFL characteristics

The characteristics of the RPC network measured in this study are in close agreement with the previous histological reports for the macaque monkey that used ink-fill to visualize RPCs [4,5]. The NFL thickness measured here also agrees with previous histological [14] and OCT [15] measures. Macaque RPCs are very similar to human RPCs, whereas those of the cat and pig are distinctly different [4]. In precise agreement with the findings of the present study, macaque and human RPCs are most prominent in the Bjerrum region that is coincident with the thickest NFL, they are of small caliber, and they are straight and elongated superficially. The characteristic parallel distribution of RPC to nerve fibers, as well as a relative lack of anastomoses, reported by Henkind [6] can be seen in Figures 2 and 3 of the present paper. Deeper images display the polygonal pattern that is typical of retinal vasculature. Figure 4 demonstrates agreement with Henkind's [4] description that RPCs are normally supplied by retinal vessels from the ganglion cell layer that arch up abruptly to sustain the NFL.

Our results clearly indicate that RPC bed thickness remains slightly less than NFL thickness, suggesting that RPC thickness in the macaque is less than that of humans and varies across the retina in parallel with NFL thickness [14,16,17]. This is a very consistent observation in all retinal sections and reflects the lack of RPCs in the superficial 10 - 20 μm of the NFL. *In-vivo *comparison of NFL and RPC bed thickness in macaques and humans would require simultaneous OCT and AO angiography.

### Potential use of RPC imaging in human eye disease

In some cases RPCs develop into collateral (bypass) vessels following retinal vein occlusions. In addition, they have been implicated as the vessels occluded which lead to cotton-wool spots (superficial NFL infarctions), associated with numerous ocular and systemic disorders. Their primary or secondary involvement in glaucoma was postulated in 1968. [6] Although potential vascular changes within the lamina cribrosa is a more likely locus for the primary site of glaucomatous damage, no currently known modality can image this region or measure its functional state. Measurement of those vascular networks which are adjacent or functionally dependent upon this vasculature would be worthy of further evaluation. The RPCs are one such vascular network which is accessible.

An important motivation for RPC imaging is to provide a better understanding and potential clinical indicator of early glaucomatous progression. As ganglion cells are lost in glaucoma the NFL progressively thins and this can be measured with high-resolution by OCT, making NFL thickness a valuable early indicator of glaucoma [18]. However, loss of NFL thickness does not provide a complete measure of glaucoma progression, since NFL thins less than the proportional loss of retinal ganglion cells [8]. One potential use of RPC imaging is to track changes in individual identified vessels, whose depth within the NFL is related to the retinal locus from which the axons originate. Thus, *in-vivo *imaging of RPCs and the surrogate blood flow indicator that they provide may complement the analysis of NFL changes during glaucomatous damage.

## Conclusion

We have demonstrated in this study that FAO-SLO techniques can deliver *in-vivo *images of RPCs and their network thicknesses with high fidelity. Application of this methodology to the measurable transverse NFL characteristics in retinal vascular or glaucoma patients is potentially feasible, since it is no more invasive than conventional angiography. One important concern is that the light exposure used in this imaging be completely safe, optimally more conservative than current ANSI safely limits for humans [19]. In other animal FAO-SLO imaging studies (data not shown) we have found that RPE damage may occur during imaging at relatively short wavelengths such as the 488 nm excitation source that is used to image fluorescein [20], but this damage can be avoided by adopting safety limits on light exposure that are more conservative than those previously used. Of additional concern is that a wider margin for exposure may be necessary, as RPE damage may occur with greater frequency or severity in the disease-affected eye [21].

## Competing interests

This research was supported in part by Bausch and Lomb, and William Merigan and Robert Wolfe were partially supported by Bausch and Lomb. The University of Rochester holds a patent on the method for adaptive optics imaging of retina.

## Authors' contributions

DS carried out AO imaging, and collected and analyzed all confocal data, DCG was involved in the design of RPC imaging, JJH, RW, YG, and BDM carried out AO imaging, BPG was involved in planning the study and writing the manuscript, RTL helped coordinate imaging and histological measures, SR was involved in writing and revising the manuscript, DRW helped plan and carry out the AO imaging study, WHM conceived the study, coordinated its execution and drafted the manuscript. All authors read and approved the final manuscript.

## Pre-publication history

The pre-publication history for this paper can be accessed here:


